# Development and Evaluation of *Platanus orientalis* L. Extract-Loaded Liposomes for Enhanced Wound Healing

**DOI:** 10.3390/ph19010032

**Published:** 2025-12-23

**Authors:** Firdevs Demirel, Ali Asram Sağıroğlu, Gülbahar Özge Alim Toraman, Aysenur Gunaydin-Akyildiz, Zehra Keskin, Beyza Sümeyye Aydın, Gülaçtı Topçu

**Affiliations:** 1 Institute of Health Sciences, İstanbul University, 34126 Istanbul, Turkey; firdevs.demirel@bezmialem.edu.tr; 2 Department of Pharmacognosy, Faculty of Pharmacy, Bezmialem Vakif University, 34093 Istanbul, Turkey; galim@bezmialem.edu.tr (G.Ö.A.T.); gtopcu@bezmialem.edu.tr (G.T.); 3 Department of Pharmaceutical Technology, Faculty of Pharmacy, Istanbul University-Cerrahpasa, 34500 Istanbul, Turkey; 4 Department of Pharmaceutical Toxicology, Faculty of Pharmacy, Istanbul University-Cerrahpasa, 34500 Istanbul, Turkey; gunaydinaysenur@gmail.com; 5 Division of Gastroenterology and Hepatology, Department of Medicine, University of Pennsylvania, Philadelphia, PA 19104, USA; 6 Department of Pharmaceutical Toxicology, Faculty of Pharmacy, Bezmialem Vakif University, 34093 Istanbul, Turkey; zehra.seker@bezmialem.edu.tr; 7 Health Sciences Institute, Bezmialem Vakif University, 34093 Istanbul, Turkey; beyza_sumeyye@hotmail.com; 8 Drug Application and Research Center (DARC), Bezmialem Vakif University, 34093 Istanbul, Turkey

**Keywords:** *Platanus orientalis*, quercetin, liposome, LC-HRMS, wound healing

## Abstract

**Background/Objectives**: Wound healing is a complex biological process influenced by inflammation, oxidative stress, and cellular regeneration. Plant-derived bioactive compounds have shown potential to accelerate tissue repair through antioxidant and anti-inflammatory mechanisms. This study aimed to develop and evaluate a *Platanus orientalis* extract-loaded liposomal formulation for potential wound-healing applications. **Methods**: Four polar extracts (P1–P4) were prepared using different solvent systems and extraction techniques and were characterized by LC-HRMS to determine their phytochemical profiles. Among the identified constituents, quercetin was consistently detected across all extracts and selected as the reference compound due to its well-known wound-healing activity. Liposomes were prepared via thin-film hydration followed by probe sonication and characterized for particle size, polydispersity index (PDI), zeta potential, encapsulation efficiency, and total drug content. In vitro release, cytotoxicity, and wound-healing assays were subsequently conducted to assess performance. **Results**: The optimized liposome formulation had a mean particle size of 106.6 ± 5.4 nm, a PDI of 0.11 ± 0.04, and a zeta potential of −14.1 ± 0.5 mV. Environmental scanning electron microscopy (ESEM) confirmed the nanosized spherical morphology and homogeneous vesicle distribution, supporting the successful development of the liposomal delivery system. Encapsulation efficiency and total drug content were determined as 72.25 ± 1.05% and 96.15 ± 0.14%, respectively. In vitro release studies demonstrated a biphasic pattern with an initial burst followed by a sustained release, reaching approximately 75% cumulative quercetin release within 24 h. Physical stability testing confirmed that the optimized liposomal formulation remained physically stable at 5 ± 3 °C for at least 60 days. The optimized formulation showed no cytotoxic effects on CDD-1079Sk fibroblast cells and exhibited significantly enhanced wound closure in vitro. **Conclusions**: These findings indicate that the liposomal delivery of *Platanus orientalis* extract provides a biocompatible and sustained-release system that enhances wound-healing efficacy, supporting its potential use in advanced topical therapeutic applications.

## 1. Introduction

The skin, being the largest organ of the body, functions primarily as a protective barrier against external insults. However, its integrity can be compromised by trauma or disease, leading to wound formation and subsequent impaired function [1]. Wound healing is a complex, intricately overlapping biological process that encompasses several distinct phases: hemostasis, inflammation, proliferation, and remodeling. During these stages, various cell types, including immune cells, fibroblasts, keratinocytes, and endothelial cells, play central and coordinated roles [2,3,4,5]. Platelets and macrophages are crucial for releasing an array of cytokines and growth factors. These molecular mediators are essential for regulating key processes such as coagulation, the recruitment of immune cells to the wound site, fibroblast proliferation, angiogenesis, and collagen deposition [3,6,7]. Recovery can be complicated and delayed by a multitude of local and systemic factors, including chronic diseases, smoking, excessive alcohol consumption, immunosuppression, certain medications, and malnutrition [1]. While conventional treatment strategies traditionally involve tissue scaffolds, antibiotics, and anti-inflammatory agents, current therapeutic approaches are increasingly focusing on the application of bioactive compounds. These compounds possess antioxidant, antimicrobial, and anti-inflammatory properties, offering protection against oxidative stress, preventing infection, and significantly accelerating tissue repair [8,9,10,11,12].

The medicinal use of plants has a long history, with their therapeutic roles differing across cultures and regions. Traditionally, various species have been employed to treat a wide range of ailments, including cardiovascular and gastrointestinal disorders, insomnia, pain, and wounds [13,14,15,16]. The pharmacological basis of many of these traditional practices has since been supported by modern scientific studies [17,18,19], and numerous ethnobotanical reports confirm that several plant species continue to be used effectively in wound management [20,21,22,23,24]. Among them, *Platanus orientalis* L. (Eastern Plane), a deciduous tree of the Platanaceae family widely distributed in Middle Eastern and Asian regions, has been traditionally used in folk medicine for its leaves, wood, and other parts [25]. Its phytochemical richness, particularly in flavonoids such as quercetin, coumarin, and kaempferol, confers antibacterial, antioxidant, anti-inflammatory, and wound-healing activities [26,27,28,29,30,31]. Extracts from *Platanus orientalis* leaves have been shown to stimulate fibroblast proliferation, enhance collagen synthesis, and promote epithelial cell growth, thereby supporting tissue regeneration [25]. Although the bioactive compounds of *Platanus orientalis* have been widely reported for their therapeutic potential, no liposomal formulation of its extract has yet been developed for topical use. In this study, quercetin, a flavonoid with well-established wound-healing properties, was chosen as the reference compound for both analytical characterization and formulation development.

Nanocarrier systems have gained considerable attention in recent years as efficient platforms for the delivery of therapeutic agents. Compared with conventional dosage forms, they offer enhanced efficacy, reduced side effects, and improved biocompatibility [32]. Among lipid-based nanocarriers, liposomes remain one of the most extensively studied systems owing to their amphiphilic bilayer structure, which enables the incorporation of both hydrophilic and lipophilic substances. Their close resemblance to biological membranes also facilitates their application to the skin, promoting dermal absorption [33]. Liposomes can penetrate the stratum corneum more effectively by squeezing through intercellular spaces, fusing with skin cell membranes, and disturbing the lipid arrangement, thereby enhancing drug permeation through diffusion and capillary action. Several approved liposomal formulations with proven clinical efficacy further validate their therapeutic potential [34,35]. Moreover, liposomes provide a versatile platform for encapsulating plant-derived bioactives, enabling the controlled and localized delivery of antioxidant, anti-inflammatory, and wound-healing compounds [36,37,38]. Polyphenol-loaded liposomes have demonstrated the ability to enhance fibroblast migration and modulate the wound microenvironment through sustained antioxidant activity and improved cellular uptake [39]. While advanced delivery platforms, including hydrogels, nanofibers, and composite wound dressings, provide structural support and promote moisture balance at the wound site, their fabrication often requires more complex processing steps and presents challenges in uniformly incorporating plant extracts [40,41,42]. Compared to these systems, liposomes offer a simpler and scalable nanocarrier approach with high compatibility for phenolic compounds [43]. However, despite the increasing interest in such systems, no liposomal formulation of *Platanus orientalis* extract has been reported previously for wound-healing applications. Therefore, the development of liposomal formulations of *Platanus orientalis* extract represents a novel and promising strategy to enhance its therapeutic potential in wound-healing applications.

We hypothesized that incorporating the *Platanus orientalis* extracts into a liposomal delivery system would provide sustained release of bioactive flavonoids such as quercetin, reduce extract-associated cytotoxicity, and consequently enhance fibroblast migration and wound-healing efficacy more effectively than the free extract. For this purpose, *Platanus orientalis* extracts were obtained using different extraction techniques and solvents with varying ethanol/water ratios to compare their phytochemical compositions and biological properties. Among the prepared extracts, the one showing the most favorable cytocompatibility and bioactivity was selected for liposomal formulation. Liposomes were then prepared using the thin-film hydration method followed by probe sonication to ensure uniform particle size distribution. In vitro characterization was conducted on liposomal formulations for optimization. The optimized formulation was subsequently evaluated for cytocompatibility and wound-healing activity through established in vitro assays.

## 2. Results and Discussion

### 2.1. Phytochemical Characterization and Cytotoxic Evaluation of Platanus orientalis Extracts

#### 2.1.1. LC/HRMS Analysis of the *Platanus orientalis* Extracts

The LC-HRMS analysis results of the polar extracts of *Platanus orientalis* (P1–P4) are presented in Table 1. Each extract was obtained from 1000 g of dried plant material using different solvent systems and extraction techniques, and analyzed both qualitatively and quantitatively by LC-HRMS. The resulting extracts were designated as P1 (ethanol—Soxhlet extract), P2 (70% ethanol—Soxhlet extract), P3 (70% ethanol—maceration extract), and P4 (ethanol—maceration extract). The chromatographic analysis revealed marked variations in the phytochemical profiles of the four extracts, indicating that both solvent polarity and extraction method significantly affected the recovery of phytochemical composition.

As shown in Table 1, the P1 extract predominantly contained chlorogenic acid, hyperoside, quercitrin, and 6-Hydroxyluteolin 7-*O*-glucoside. The P2 extract was rich in ascorbic acid, chlorogenic acid, fumaric acid, rutin, quercitrin, quercetin, rhamnocitrin, and hispidulin-7-glucoside. In the P3 extract, rutin, hyperoside, quercitrin, quercetin, and hispidulin-7-glucoside were the major constituents, while the P4 extract contained high levels of ascorbic acid, chlorogenic acid, quercitrin, quercetin, rhamnocitrin, hispidulin-7-glucoside, and 6-Hydroxyluteolin 7-*O*-glucoside. The concentrations of these bioactive compounds differed notably among the extracts, reflecting the strong influence of extraction parameters on phytochemical yield and composition.

Among the identified flavonoids, quercetin was consistently detected in all *Platanus orientalis* extracts (Table 2). Owing to its well-documented pharmacological propertiesparticularly its ability to modulate inflammatory pathways, stimulate fibroblast proliferation, and promote collagen synthesis, quercetin was selected as the reference compound for comparative evaluation [44,45,46]. Given its recognized role in the wound-healing process, quercetin serves as a reliable indicator of the therapeutic potential of *Platanus orientalis* extracts. Furthermore, the majority of both major and minor constituents exhibited antioxidant, anti-inflammatory, and cytoprotective activities, suggesting that their combined presence may contribute synergistically to the overall wound-healing efficacy of the extract [44,47,48,49,50,51,52,53].

#### 2.1.2. MTT Cytotoxicity Results of *Platanus orientalis* Extracts

**Figure 1:**
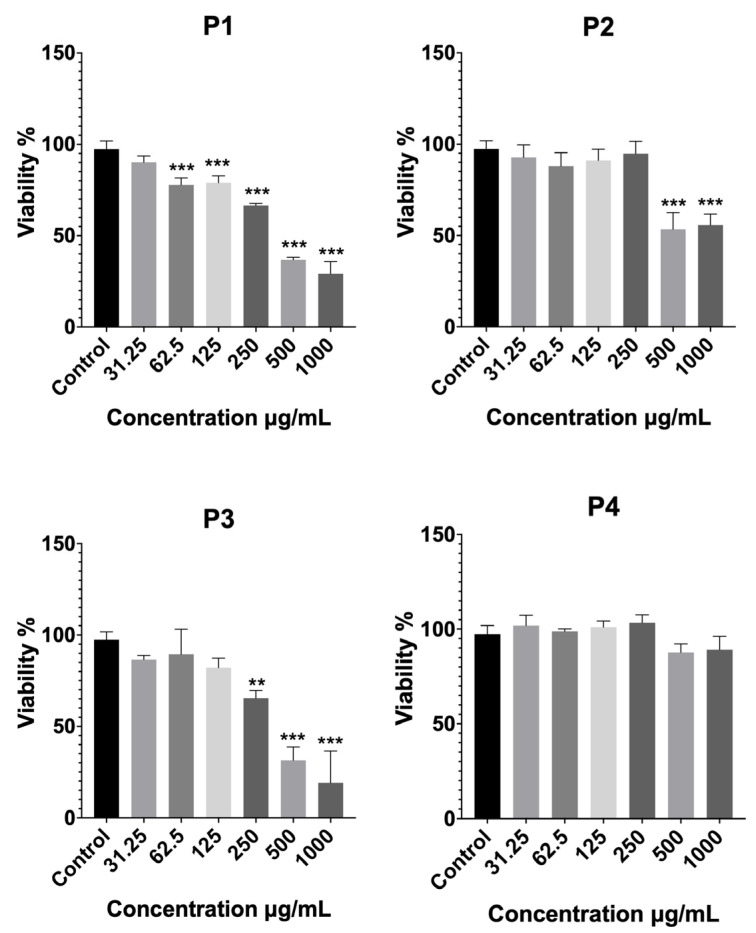
Cytotoxicity profiles of *Platanus orientalis* extracts (P1–P4), determined by MTT assay after treatment for 24 h on fibroblast cells. Data are expressed as mean ± SD. Statistical comparisons were carried out using one-way ANOVA followed by Tukey’s post hoc test, ** *p* < 0.01, and *** *p* < 0.001 versus the control group. P1 (ethanol—Soxhlet extract), P2 (70% ethanol—Soxhlet extract), P3 (70% ethanol—maceration extract), and P4 (ethanol—maceration extract).

The cytotoxic potential of the four *Platanus orientalis* extracts (P1–P4) was assessed on fibroblast cells using the MTT assay, and the results are shown in Figure 1. Extracts P1 and P3 exhibited a clear dose-dependent cytotoxic effect, with cell viability decreasing sharply at concentrations ≥250 µg/mL. At the highest tested concentration (1000 µg/mL), both extracts reduced cell viability to below 30%, indicating pronounced cytotoxicity. Similarly, P2 caused a marked reduction in viability at concentrations of 500 µg/mL and above, with cell survival decreasing by nearly 50% relative to the control. These results suggest that the P1, P2, and P3 extracts contain bioactive constituents with potential cytotoxic and antiproliferative effects, making them unsuitable for further development in wound-healing formulations.

In contrast, the P4 extract maintained high cell viability across the entire concentration range (31.25–1000 µg/mL), with no statistically significant difference from the control group. A slight, non-significant decrease was observed only at the two highest concentrations. This demonstrates its excellent biocompatibility and lack of cytotoxic effects. From a formulation standpoint, such cytocompatibility is essential for wound-healing applications, where safe and non-toxic systems are required to support fibroblast proliferation and tissue regeneration.

Overall, the cytotoxicity evaluation clearly identified P4 as the most biocompatible extract, exhibiting superior cell tolerance compared with the other extracts (P1, P2, and P3). Consequently, P4 was selected for subsequent liposomal formulation development and in vitro activity evaluation, based on its favorable safety and therapeutic profile.

### 2.2. Preparation and Characterization of Liposome Formulations

#### 2.2.1. Particle Size, Polydispersity Index (PDI) and Zeta Potential Analyses

To optimize the liposomal formulations, six different compositions were prepared by varying the ratios of phospholipid (50, 100, and 150 mg) and cholesterol (5, 10 mg) (Table 3). Dynamic light scattering analysis revealed that both phospholipid and cholesterol concentrations affected particle size, PDI, and zeta potential values.

When phospholipid content was varied while keeping cholesterol levels constant (5 or 10 mg), clear differences were observed in the physicochemical behavior of the liposomes. In both cholesterol groups, lipid composition had a marked influence on vesicle size and uniformity. At 5 mg cholesterol, increasing phospholipid from 50 to 150 mg first caused a slight rise in particle size and then a sharp decrease at the highest lipid level. This reduction was accompanied by a narrower size distribution and a more negative zeta potential, suggesting that higher phospholipid content favors the formation of compact, well-organized bilayers and improves colloidal stability.

In contrast, when cholesterol was increased to 10 mg, the effect of additional phospholipid was less favorable. Although particle size decreased slightly from F5 to F6, the PDI values increased, pointing to greater heterogeneity within the vesicle population. The excess cholesterol likely made the bilayer more rigid, limiting its ability to break down into small, uniform particles during sonication and promoting partial aggregation instead.

Overall, higher phospholipid concentrations tended to improve size uniformity and surface stability, whereas high cholesterol content reduced membrane flexibility and homogeneity. Among all formulations, F3 displayed the most balanced physicochemical profile, combining a nanoscale particle size with low PDI and adequate surface charge for stability. Therefore, this formulation was identified as the optimum extract-loaded liposomal formulation, providing a suitable carrier system for further characterization and wound-healing studies.

#### 2.2.2. Encapsulation Efficiency

The encapsulation efficiency of quercetin was determined using the centrifugation method followed by quantitative LC-HRMS analysis. The optimized liposomal formulation (F3) achieved an encapsulation efficiency of 72.25 ± 1.05%, indicating that a large fraction of quercetin was successfully incorporated within the lipid bilayer. This high efficiency can be attributed to the lipid composition of F3, where a higher phospholipid content combined with a lower cholesterol ratio created a favorable microenvironment for the entrapment of quercetin. Similar observations have been reported for quercetin-loaded liposomes, in which strong hydrophobic interactions between quercetin molecules and the lipid bilayer facilitated efficient encapsulation and contributed to a sustained release profile [54]. Overall, these findings highlight the suitability of the developed liposomal system for efficient loading of *Platanus orientalis* bioactives and provide a solid foundation for its further evaluation in wound-healing applications.

#### 2.2.3. Drug Content

The drug content of the optimum liposomal formulation was evaluated to determine the proportion of quercetin retained within the liposomal system. Following complete disruption of the vesicles with methanol and subsequent LC-HRMS quantification, the drug content was calculated as 96.15 ± 0.14%. This high drug content value demonstrates that nearly all of the quercetin incorporated during the formulation process was successfully retained within the liposomal bilayer, with minimal loss during preparation. The result reflects both the compatibility of quercetin with the lipid matrix and the efficiency of the selected formulation method.

#### 2.2.4. Morphology Studies

**Figure 2:**
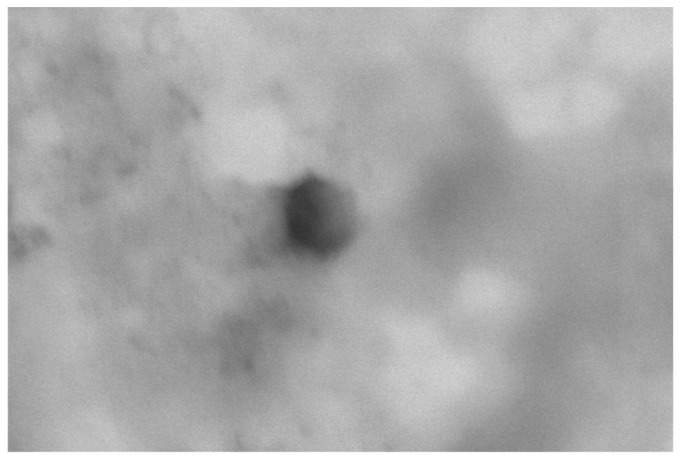
ESEM images of the optimized liposome (scale bar: 300 nm).

Verification of particle size and morphological characteristics is a critical component in the comprehensive evaluation of liposomal systems. In this study, particle size measurements obtained by dynamic light scattering were supported by microscopic inspection to ensure the reliability of the results. The consistency between the particle size distribution and the observed vesicular morphology confirms the accuracy of the measurements, as illustrated in Figure 2. Microscopic visualization demonstrated that the optimized formulation consisted of nanosized, spherical vesicles with smooth surfaces.

#### 2.2.5. In Vitro Release Studies

**Figure 3:**
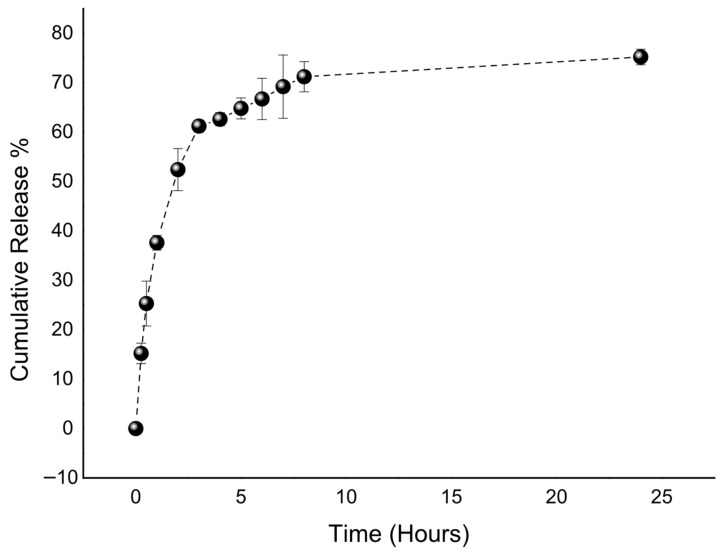
In vitro release profile of the optimized liposome.

The release profile of quercetin from the optimized liposomal formulation was monitored over 24 h (Figure 3). The data revealed a characteristic biphasic pattern, consisting of an initial burst release followed by a slower, sustained phase. Approximately 60% of the quercetin was released within the first 4 h, after which the rate of release gradually declined, reaching about 75% at 24 h. This pattern is likely explained by two concurrent mechanisms, where quercetin bound to the surface or loosely associated with the vesicles is released rapidly at the early stage, while the drug entrapped within the lipid bilayers is released more slowly in a diffusion-controlled manner. The sustained phase can be attributed to the stabilizing influence of phospholipids and cholesterol, which reinforce the bilayer structure and slow the diffusion of quercetin into the surrounding medium. Consistent with these findings, another study on quercetin-loaded liposomes for wound-healing applications also demonstrated a comparable biphasic release pattern, with an initial burst phase followed by sustained diffusion-mediated release, supporting the reproducibility of this release behavior across similar liposomal systems [55]. A moderate burst release, as observed here, can be particularly advantageous in wound-healing applications, providing an immediate therapeutic level of antioxidant and anti-inflammatory activity while maintaining prolonged drug availability at the target site.

#### 2.2.6. Stability Studies

Stability studies for the optimized liposomal formulation were performed under refrigerated storage conditions at 5 ± 3 °C for 60 days. Samples were collected at predetermined intervals, and particle size, PDI, zeta potential, and physical appearance were evaluated. The measured particle size, PDI, and zeta potential values during the storage period are presented in Table 4. No statistically significant changes were observed over time (*p* > 0.05), indicating that the optimized liposomal formulation maintained its physicochemical stability for at least two months under refrigerated conditions.

### 2.3. Bioactivity of the Optimized Extract-Loaded Liposomal Formulation

#### 2.3.1. Cytotoxicity Results

**Figure 4:**
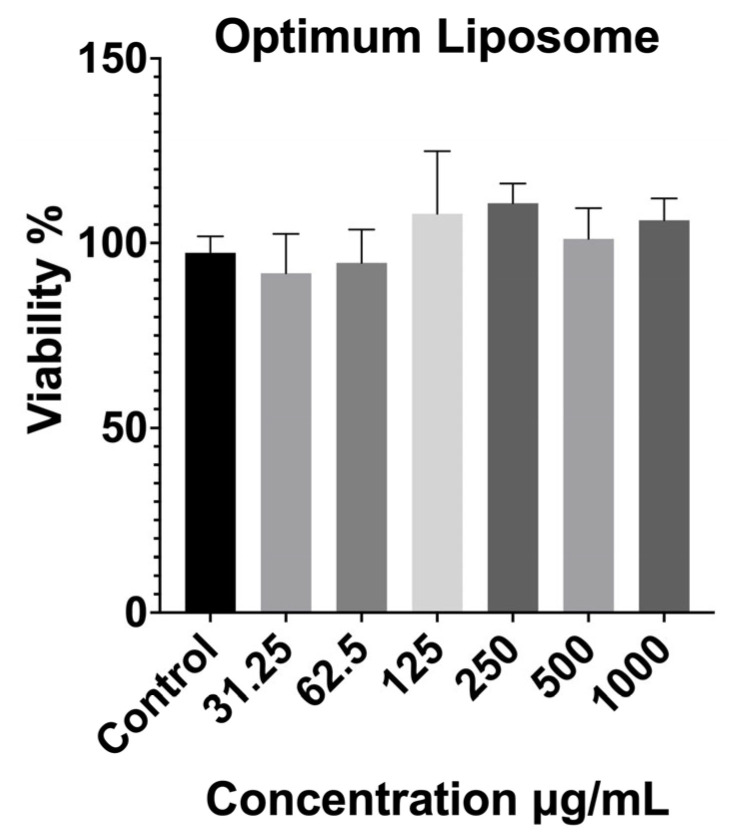
Cytotoxicity results of the optimized liposomal formulation on fibroblast cells after 24 h exposure, determined by MTT assay. Data are expressed as mean ± SD. Statistical comparisons were carried out using one-way ANOVA followed by Tukey’s post hoc test compared with the control group.

The cytocompatibility of the optimized extract-loaded liposomal formulation (F3) was evaluated using the MTT assay at concentrations ranging from 31.25 to 1000 µg/mL (Figure 4). The results showed that F3 maintained high cell viability across all tested concentrations, with no statistically significant reduction compared to the untreated control group. Even at the highest concentration (1000 µg/mL), cell viability exceeded 90%, confirming the absence of dose-dependent cytotoxic effects.

These findings indicate that incorporating the optimized extract-loaded liposomal carrier did not introduce additional cytotoxicity and that the resulting formulation was well tolerated by the fibroblast cells. The excellent biocompatibility of F3 may be attributed to both the mild extraction conditions used for P4, which produced a phytochemical profile rich in glycosides and less reactive flavonoid derivatives, and the protective nature of the liposomal bilayer, which can shield cells from direct contact with potentially irritant components. From an application standpoint, the negligible cytotoxicity of the optimized liposome is a key advantage for wound healing and dermal therapies, where formulation safety and cellular compatibility are essential prerequisites for clinical translation.

#### 2.3.2. Cell Migration and Proliferation Assay

**Figure 5:**
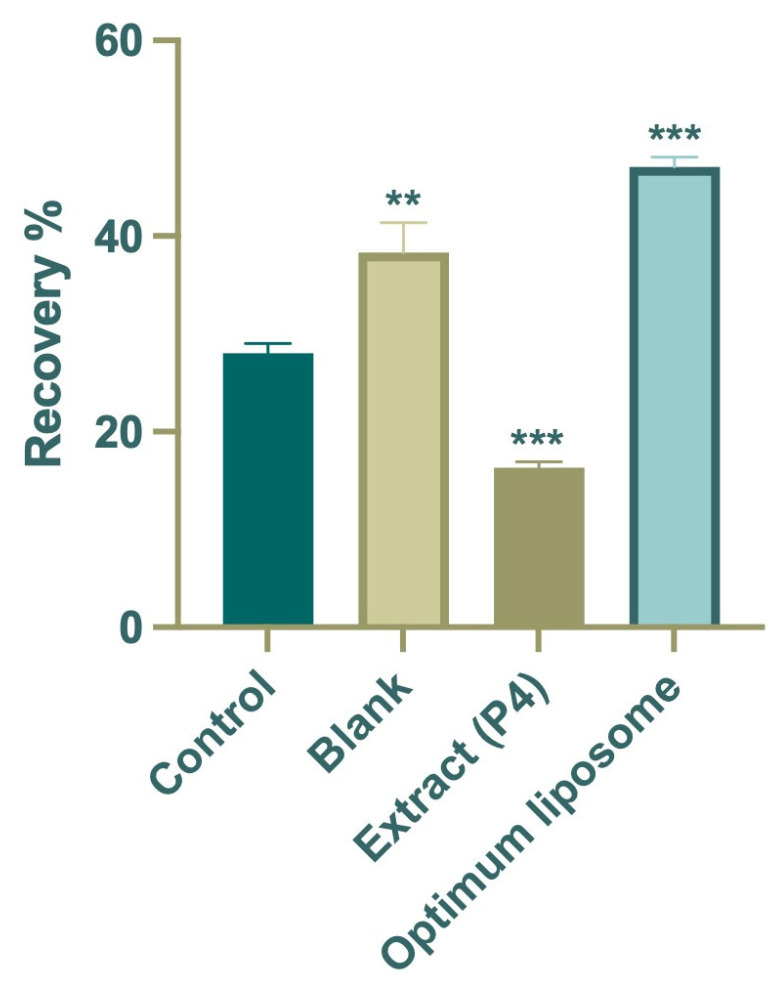
Average percentage wound-healing rates of the extract (P4), optimum formulation (F3), blank, and control groups. Data are expressed as mean ± SD. Statistical comparisons were carried out using one-way ANOVA followed by Tukey’s post hoc test, ** *p* < 0.01 and *** *p* < 0.001 versus the control group.

The wound-healing assay was performed to evaluate the influence of the P4 extract and its optimized liposomal formulation on fibroblast cell migration, a key event in the tissue repair process. The results are shown in Figure 5. After 24 h of incubation, the control group exhibited a moderate wound closure of approximately 28%, which reflects the normal migratory behavior of untreated cells. In comparison, cells treated with the free P4 extract showed lower migration (~16%), suggesting that the extract alone was not sufficient to promote wound closure, likely due to the limited solubility and cellular uptake of its bioactive components.

**Figure 6:**
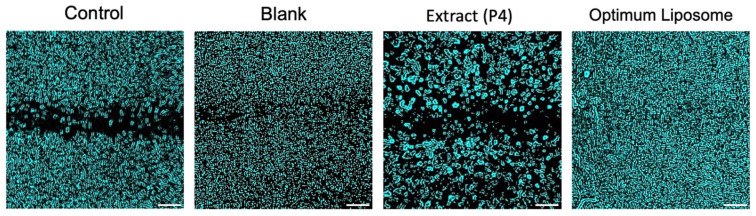
In vitro wound-healing assay. Images of cell migration for control, blank, extract (P4), and optimized liposomal formulation at 0 h and 24 h after treatment (Scale bar: 50 µm).

In contrast, the optimized liposomal formulation (F3) markedly enhanced cell migration, achieving nearly 47% wound closure after 24 h, almost double that of the control group and significantly higher than the extract-treated cells. Microscopic images taken before and after exposure (Figure 6) supported these quantitative findings, clearly showing more extensive closure of the scratch area in the F3-treated wells. The improved wound-healing activity observed with F3 may be associated with the delivery of *Platanus orientalis* bioactives through the liposomal formulation, which appeared to promote fibroblast migration more effectively than the free extract. The protective lipid bilayer likely facilitates prolonged exposure of cells to active flavonoids such as quercetin, enabling more effective stimulation of migration and repair. These findings align with earlier reports indicating that nanoencapsulation of plant-derived polyphenols enhances their therapeutic performance in wound-healing models by improving cellular uptake and maintaining effective local concentrations [56,57,58].

The enhanced wound-healing performance observed for the extract-loaded liposomal formulation can be attributed to multiple complementary mechanisms. The nanoscale vesicle size is expected to facilitate enhanced cellular internalization and more efficient interaction with the wound environment. In addition, the sustained release of encapsulated phytochemicals likely maintains prolonged local exposure to bioactive compounds with antioxidant and anti-inflammatory activities. Encapsulation may improve the stability and delivery efficiency of the bioactive compounds, enhancing their ability to modulate cytokine-regulated pathways that promote fibroblast migration and ultimately contribute to faster wound closure [59,60].

In summary, while the free P4 extract exhibited limited activity, its incorporation into the liposomal carrier system (F3) significantly improved the wound closure rate and overall healing potential. These results underscore the advantage of nanocarrier-based delivery in enhancing the therapeutic efficacy of plant-derived bioactives and identify F3 as a promising candidate for future preclinical wound-healing studies.

Despite the promising findings, this study has certain limitations that should be considered. The biological evaluation was limited to a single fibroblast cell line, which reflects only one aspect of the wound-healing process. More comprehensive assessments, including additional mechanistic endpoints and advanced wound models, are needed to further elucidate the therapeutic potential of the optimized liposomal formulation. Future work will therefore focus on expanding the dose–response evaluation and confirming the results in in vivo systems.

## 3. Materials and Methods

### 3.1. Materials

Plant materials of *Platanus orientalis* L. were collected from Buyukcekmece in Istanbul at 41°04′27.5″ N 28°37′13.5″ E. Plant materials were dried under controlled conditions at 15–25 °C in a well-ventilated, dark environment with ambient humidity below 40%. Phospholipid (containing 95% phosphatidylcholine from soybean) was obtained from Lipoid AG (Steinhausen, Switzerland). Cholesterol was purchased from Sigma-Aldrich (St. Louis, MO, USA). All solvents used for extraction and analytical procedures were supplied by Merck Chemicals Ltd. (Darmstadt, Germany). CCD-1079Sk normal human dermal fibroblast cells were obtained from the American Type Culture Collection (ATCC, Manassas, VA, USA). All reagents and chemicals used in this study were of analytical grade.

### 3.2. Methods

#### 3.2.1. Preparation and Characterization of *Platanus orientalis* Extracts

##### Extraction of the Plant Materials

A total of 1000 g of dried and shredded *Platanus orientalis* leaves was divided into four equal portions (250 g) to prepare extracts using different extraction methods. Two portions were subjected to Soxhlet extraction for two days using ethanol and 70% ethanol as solvents, respectively. The remaining two portions were extracted by maceration with ethanol and 70% ethanol, and the extraction was conducted by maceration for 72 h, repeated five times. All obtained extracts were concentrated under reduced pressure using a rotary evaporator. Finally, four different extracts (P1-EtOH—Soxhlet extract, P2—70% ethanol—Soxhlet extract, P3—70% ethanol—maceration extract, P4—ethanol—maceration extract) were obtained and analyzed with LC-HRMS.

##### LC/HRMS Analysis of the *Platanus orientalis* Extracts

The phytochemical composition of the *Platanus orientalis* extracts (P1–P4) was investigated by liquid chromatography coupled with high-resolution mass spectrometry (LC-HRMS) [61,62,63]. Chromatographic separation was achieved on a reversed-phase Troyasil C18 column (150 × 2.1 mm, 1.7 μm particle size) maintained at 40 °C. The mobile phases consisted of solvent A (0.1% formic acid in water, *v*/*v*) and solvent B (0.1% formic acid in methanol, *v*/*v*). The gradient program was applied as follows: 0.00–1.00 min, 50% B; 1.00–3.00 min, 50–100% B; 3.00–6.00 min, 100% B; 6.00–7.00 min, 100–50% B; and 7.00–15.00 min, 50% B for re-equilibration, at a constant flow rate of 0.35 mL/min with an injection volume of 5 μL.

Mass spectrometric analysis was conducted on a Thermo Orbitrap Q-Exactive system equipped with an electrospray ionization (ESI) source, operating in both positive and negative ion modes across the *m*/*z* range of 100–900. The optimized MS parameters were as follows: sheath gas flow rate, 45; auxiliary gas flow rate, 10; spray voltage, 3.80 kV; capillary temperature, 320 °C; auxiliary gas heater temperature, 320 °C; and S-lens RF level, 50. Phenolic compounds were identified by comparing retention times and high-resolution MS data with those of authentic standards.

##### MTT Cytotoxicity Assay of *Platanus orientalis* Extracts

The cytotoxicity of the four *Platanus orientalis* extracts (P1–P4) was evaluated using the MTT assay, in order to determine their biocompatibility and select the most suitable extract for further formulation studies. Briefly, Fibroblast cells were seeded into 96-well plates and treated with serial dilutions of each extract, starting from 1000 µg/mL and diluted 1:1 across the plate. After 24 h incubation at 37 °C, 20 μL of MTT solution (5 mg/mL) was added to each well, and the plates were incubated for an additional 3 h under dark conditions. The supernatant was then removed, and dimethyl sulfoxide (DMSO) was added to dissolve the formazan crystals.

Absorbance was measured at 590 nm using a BioTek Synergy H1 microplate reader (Epoch, Nuremberg, Germany). Cell viability was calculated relative to the untreated control group. Data are expressed as mean ± SD of three independent experiments, and statistical analysis was performed using one-way ANOVA (* *p* < 0.05, vs. control).

#### 3.2.2. Preparation and Characterization of Formulations

##### Preparation of Liposomal Formulations

Liposomes were prepared by the thin-film hydration method [55,64,65]. Phospholipid and cholesterol were dissolved in chloroform at the ratios indicated in Table 5. The solvent was evaporated under reduced pressure at 40 °C using a rotary evaporator to obtain a thin lipid film on the flask wall. The film was then hydrated with an aqueous solution of selected *Platanus orientalis* extract prepared in distilled water, followed by gentle agitation to form multilamellar vesicles. To obtain uniformly sized unilamellar liposomes, the suspensions were subjected to probe sonication using a tip ultrasonicator (40% amplitude, 5 min). The optimum formulation was determined based on particle size and PDI values.

##### Determination of Particle Size, Polydispersity Index (PDI), and Zeta Potential

Particle size and PDI sizes were determined by a dynamic light scattering method using ZetaSizer Nano ZSP (Malvern Instruments Ltd., Malvern, UK). Zeta potential values were measured by the Laser Doppler micro electrophoresis method using special cuvettes (Malvern Instruments Ltd.). For this purpose, liposome suspensions were diluted (1:100) with deionized water to prevent multiple scattering effects. All measurements were performed at room temperature and were repeated at least three times.

##### Determination of Encapsulation Efficiency

Encapsulation efficiency of quercetin was determined by the centrifugation method followed by quantitative LC-HRMS analysis. Briefly, 1 mL of each liposomal formulation was centrifuged at 15,000 rpm for 30 min at 4 °C to separate the unencapsulated quercetin from the encapsulated fraction. Unencapsulated quercetin was analyzed using the LC-HRMS method described above. Encapsulation efficiency was calculated according to the following equation [66,67,68].

$$Encapsulation\;Efficiency\;\%=\frac{the\;amount\;of\;total\;drug-the\;amount\;of\;non-encapsulated\;drug}{the\;amount\;of\;total\;drug}\times 100$$

##### Total Drug Content of Liposomes

In order to determine the drug content of the formulations, 100 μL of the liposome sample was mixed with methanol and vortexed to ensure complete dissolution of the lipid layer and quercetin. The obtained solution was appropriately filtered through a 0.45 μm membrane filter. Quantitative determination of quercetin was performed using the LC-HRMS system described above. The total drug content was calculated as the percentage of the experimentally determined quercetin amount relative to the theoretical amount incorporated into the formulation.

##### Morphology Studies

The morphological properties of the optimized liposomal formulation were examined using a Thermo Fisher Quattro Environmental Scanning Electron Microscope, Waltham, MA, USA (ESEM). Prior to analysis, the liposome dispersion was diluted with deionized water to avoid particle aggregation. A small volume of the diluted sample was carefully placed onto a carbon-coated copper grid and allowed to dry at room temperature to obtain a thin film. The dried samples were then imaged under high vacuum at an accelerating voltage of 30 kV.

##### In Vitro Release Study

The in vitro release of quercetin from the optimized liposomal formulation was evaluated using the dialysis membrane method [68]. An aliquot of 1 mL of the liposome dispersion was placed into a pre-soaked dialysis bag (molecular weight cut-off: 12–14 kDa) and immersed in 150 mL of phosphate-buffered solution (PBS, pH 7.4) containing 20% ethanol to maintain sink conditions. The system was maintained at 32 ± 0.5 °C under constant stirring (100 rpm) throughout the experiment. At predetermined time intervals (0, 0.25, 0.5, 1, 2, 3, 4, 5, 6, 7, 8, and 24 h), 1 mL of the release medium was withdrawn and replaced with an equal volume of fresh buffer to maintain sink conditions. Samples were diluted with methanol (50:50, *v*/*v*) prior to LC-HRMS analysis. The diluted solutions were filtered through 0.45 μm membrane filters and analyzed by the validated LC-HRMS method described above to determine the concentration of quercetin released at each time point.

##### Stability Study

For stability evaluation of the optimized liposomal formulation, samples were stored at 5 ± 3 °C and monitored over a period of 2 months. At predetermined intervals, the formulations were analyzed for particle size, PDI, zeta potential, and physical appearance to assess potential changes in physicochemical properties. All measurements were performed in triplicate.

#### 3.2.3. Cell Culture Studies

##### MTT Cytotoxicity Assay

The cytotoxicity of the optimized liposomal formulation was evaluated using the MTT assay to assess its biocompatibility at different concentrations [69,70,71]. Briefly, cells were seeded into 96-well plates and treated with serial dilutions of the liposomal suspension, starting from 1000 µg/mL and applying 1:1 serial dilutions across the plate. Following 24 h incubation at 37 °C under a humidified atmosphere, 20 μL of MTT solution (5 mg/mL) was added to each well, and the plates were further incubated for 3 h in the dark. After removing the supernatant, DMSO was added to the wells to dissolve the formazan crystals. Optical density was measured with BioTek Synergy H1 (Epoch, Germany) microplate reader at 590 nm. Cell viability was calculated as a percentage of the control group.

##### Cell Migration and Proliferation Assay

The CytoSelect™ 24-Well Wound-Healing Assay Kit (Cell Biolabs Inc., San Diego, CA, USA) was used to evaluate the wound-healing activity of the fibroblast cells cultured in serum-containing media to evaluate an overall in vitro wound-healing effect, which involves both cell migration and proliferation. Concentrations below cytotoxic levels were determined from the results of the MTT assay, and 250 µg/mL was selected as the test concentration due to its lower toxicity. The study included four groups, namely control (untreated cells), blank (empty liposomal formulation), *Platanus orientalis* extract (P4), and optimized extract-loaded liposomal formulation (F3) [70,72,73]. Briefly, 500 μL of medium containing 2 × 105 cells was added to the gaps of the inserts. After 24 h in 37 °C incubation for the cells to provide a monolayer, the inserts were removed, and the images of each well were taken before treatment. Treatment was done for 24 h. Images of the wound area were captured immediately after removing the inserts (0 h) and after 24 h of exposure. The study was done in triplicate (biological and technical). The wound area was quantified using ImageJ software version 1.54p (finding edges and analyzing particles), and the percentage of wound closure was calculated relative to the initial wound area according to the following equation [74].

Wound-healing area (%) = (Wt_0_ − Wt_24_)/Wt_0_ × 100

where Wt_0_ is the percentage of the cell-free area at the initial time, and Wt_24_ is the percentage of the cell-free area 24 h after post-treatment.

#### 3.2.4. Statistical Analysis

All analyses were performed in at least triplicate, and the results are expressed as mean ± standard deviation (SD). Statistical comparisons were carried out using one-way ANOVA followed by Tukey’s post hoc test, with differences considered statistically significant at *p* < 0.05. Prism 10 software was used for analysis and graphics.

## 4. Conclusions

In this study, *Platanus orientalis* leaf extracts were prepared using different extraction techniques and solvent systems, and their phytochemical profiles were characterized by LC-HRMS. Among the four extracts, P4 showed the best cytocompatibility and was therefore selected for formulation studies. Liposomal systems were successfully developed and optimized using varying ratios of phospholipid and cholesterol, with the F3 composition exhibiting the most favorable physicochemical properties. The optimized extract-loaded liposomal formulation demonstrated high encapsulation efficiency and drug content, along with a biphasic release profile providing both rapid and sustained delivery of quercetin. ESEM analysis validated spherical and uniform vesicle morphology, confirming the structural integrity of the formulation. An optimized liposome was found to be stable for 60 days at a storage temperature of 5 ± 3 °C. Cell culture studies confirmed the excellent biocompatibility of the optimized liposome formulation, while the wound-healing assay revealed a significant enhancement in fibroblast migration compared with the control group. These results indicate that the liposomal delivery system markedly improves the biological efficacy of *Platanus orientalis* bioactives. Overall, this work highlights the potential of *Platanus orientalis* extract-loaded liposomes as a promising nanocarrier platform for wound-healing applications. These findings lay a solid scientific foundation for subsequent in vivo investigations and the rational design of nanotechnology-based therapeutic systems derived from natural bioactives.

## Figures and Tables

**Table 1:** Quantitative LC–HRMS results and relative uncertainty (R.U.) values of phytochemicals identified in *Platanus orientalis* extracts (P1–P4).

Component	P1 (mg/kg)	R.U. (%)	P2 (mg/kg)	R.U. (%)	P3 (mg/kg)	R.U. (%)	P4 (mg/kg)	R.U. (%)
Ascorbic acid	398.46	3.94	7505.85	3.94	213.07	3.94	437.75	3.94
(−)-Epigallocatechin	0.71	3.09	-	-	-	-	0.19	3.09
(+)-Catechin	1.11	3.31	-	-	-	-	-	-
Chlorogenic acid	3351.05	3.58	55,044.95	3.58	-	-	4106.56	3.58
Fumaric acid	-	-	30,167.09	2.88	-	-	-	-
Caffeic acid	24.55	3.74	955.38	3.74	107.29	3.74	29.67	3.74
(+)-trans Taxifolin	4.16	3.35	4.84	3.35	13.03	3.35	4.43	3.35
Luteolin-7-*O*-rutinoside	-	-	1033.66	3.06	41.83	3.06	-	-
Naringin	-	-	425.97	4.20	22.99	4.20	13.44	4.20
Rutin	763.41	3.07	34,167.92	3.07	1828.96	3.07	1032.74	3.07
Hyperoside	7678.34	3.46	231,277.94	3.46	10,195.66	3.46	10,924.35	3.46
Dihydrokaempferol	4.28	2.86	61.20	2.86	13.11	2.86	5.01	2.86
Quercitrin	1601.74	3.78	41,843.00	3.78	2125.22	3.78	2272.74	3.78
Myricetin	118.26	4.18	3038.09	4.18	298.84	4.18	71.15	4.18
Quercetin	263.84	2.95	7504.99	2.95	920.40	2.95	209.90	2.95
Salicylic acid	96.63	1.89	2436.51	1.89	120.36	1.89	127.42	1.89
Naringenin	19.29	4.20	372.76	4.20	40.52	4.20	25.17	4.20
Nepetin	79.92	2.19	1773.55	2.19	112.35	2.19	96.89	2.19
Kaempferol	103.64	3.56	1715.01	3.56	139.84	3.56	48.00	3.56
Rhamnocitrin	654.02	3.16	16,990.77	3.16	828.96	3.16	910.72	3.16
Chrysin	4.44	3.24	140.41	3.24	7.77	3.24	7.57	3.24
Acacetin	97.82	3.98	2392.50	3.98	135.74	3.98	132.31	3.98
Hispidulin-7-glucoside	723.09	4.57	19,561.99	4.57	1203.35	4.57	989.36	4.57
Pinocembrin	5.35	3.28	215.91	3.28	8.33	3.28	7.15	3.28
Genkwanin	7.64	4.44	198.61	4.44	12.43	4.44	11.34	4.44
6-Hydroxyluteolin 7-*O*-glucoside	3531.21	2.99	-	-	-	-	5276.93	2.99
Apigenin-7-*O*-methyl ether	12.40	2.94	265.10	2.94	17.05	2.94	18.17	2.94

**Table 2:** Quercetin amounts in all extracts.

Extract Code	Quercetin Quantities (mg/kg)
P1	263.84
P2	7504.99
P3	920.40
P4	209.91

**Table 3:** Effect of phospholipid and cholesterol ratio on the particle size, PDI, and zeta potential of liposomal formulations.

Formulation	Particle Size (nm)	Polydispersity Index (PDI)	Zeta Potential (mV)
F1	270.3 ± 6.1	0.24 ± 0.12	−12.4 ± 0.6
F2	483.2 ± 11.2	0.46 ± 0.13	−12.2 ± 0.3
F3	106.6 ± 5.4	0.11 ± 0.04	−14.1 ± 0.5
F4	270.6 ± 9.5	0.32 ± 0.16	−9.18 ± 0.3
F5	415.7± 8.5	0.31 ± 0.12	−10.2 ± 0.7
F6	321.3 ± 12.5	0.56 ± 0.09	−11.2 ± 0.2

**Table 4:** The results of stability studies of optimum liposomes.

Time (Days)	Particle Size (nm)	Zeta Potential (−mV)	PDI
0	106.6 ± 5.4	14.1 ± 0.5	0.11 ± 0.04
30	112.5 ± 2.5	13.2 ± 0.6	0.14 ± 0.06
60	109.3 ± 2.2	11.4 ± 0.3	0.16 ± 0.03

**Table 5:** Composition of liposomal formulations prepared with different phospholipid and cholesterol ratios.

Code Name	Phospholipid	Cholesterol
F1	50	5
F2	100	5
F3	150	5
F4	50	10
F5	100	10
F6	150	10

## Data Availability

The data used to support the findings of this study are available from the corresponding author upon request.

## Abbreviations

The following abbreviations are used in this manuscript:

PDI	Polydispersity Index
R.U.	Relative Uncertainty
ESI	Electrospray Ionization
DMSO	Dimethyl Sulfoxide
PBS	Phosphate Buffer Solution
ESEM	Environmental Scanning Electron Microscope
